# Pharmacologic activation of autophagy without direct mTOR inhibition as a therapeutic strategy for treating dry macular degeneration

**DOI:** 10.18632/aging.202974

**Published:** 2021-04-19

**Authors:** Qitao Zhang, Feriel Presswalla, Robin R. Ali, David N. Zacks, Debra A. Thompson, Jason ML. Miller

**Affiliations:** 1 Kellogg Eye Center, University of Michigan, Ann Arbor, MI 48105, USA; 2 KCL Centre for Cell and Gene Therapy, London, England WC2R 2LS, United Kingdom; 3 Department of Biological Chemistry, University of Michigan, Ann Arbor, MI 48105, USA

**Keywords:** age-related macular degeneration (AMD), drusen, lipofuscin, retinal pigment epithelium (RPE), autophagy

## Abstract

Dry age-related macular degeneration (AMD) is marked by the accumulation of extracellular and intracellular lipid-rich deposits within and around the retinal pigment epithelium (RPE). Inducing autophagy, a conserved, intracellular degradative pathway, is a potential treatment strategy to prevent disease by clearing these deposits. However, mTOR inhibition, the major mechanism for inducing autophagy, disrupts core RPE functions. Here, we screened autophagy inducers that do not directly inhibit mTOR for their potential as an AMD therapeutic in primary human RPE culture. Only two out of more than thirty autophagy inducers tested reliably increased autophagy flux in RPE, emphasizing that autophagy induction mechanistically differs across distinct tissues. In contrast to mTOR inhibitors, these compounds preserved RPE health, and one inducer, the FDA-approved compound flubendazole (FLBZ), reduced the secretion of apolipoprotein that contributes to extracellular deposits termed drusen. Simultaneously, FLBZ increased production of the lipid-degradation product β-hydroxybutyrate, which is used by photoreceptor cells as an energy source. FLBZ also reduced the accumulation of intracellular deposits, termed lipofuscin, and alleviated lipofuscin-induced cellular senescence and tight-junction disruption. FLBZ triggered compaction of lipofuscin-like granules into a potentially less toxic form. Thus, induction of RPE autophagy without direct mTOR inhibition is a promising therapeutic approach for dry AMD.

## INTRODUCTION

Age-related macular degeneration (AMD), the leading cause of irreversible blindness in the developed world, is marked histologically by the accumulation of lipid-rich deposits in and around the retinal pigment epithelium (RPE). Intracellular lipid-rich accumulations are termed lipofuscin while extracellular accumulations are termed drusen [1, 2]. The RPE is a polarized monolayer, facing a fenestrated capillary bed termed the choroid basolaterally and a photoreceptor cell layer apically. The RPE is a high-volume consumer of lipid via uptake of lipoprotein particles from the choroidal circulation and daily ingestion of lipid-rich outer segments (OS) from photoreceptor cells. The RPE is also a prolific lipid secretor, with lipoprotein particles directed apically providing lipid for photoreceptor OS synthesis and lipoprotein particles directed basolaterally sending unneeded lipid through Bruch’s membrane to the choroid [3]. The accumulation of intracellular lipofuscin in AMD is likely the result of inefficient breakdown of phagocytosed OS [4, 5], while the accumulation of extracellular drusen likely derives from lipoprotein particles secreted by the RPE and trapped in the underlying basement membrane [3]. Improving the RPE’s capacity for lipid handling may alleviate each of these histologic hallmarks of AMD.

Autophagy is a major cellular mechanism for degrading both molecules and organelles. A *de novo* double-membrane autophagosome engulfs target cargo and then fuses with the lysosome to promote degradation of the engulfed content. Autophagy has been implicated in degradation of insoluble pathologic aggregates in neurodegenerative diseases [6] and intracellular lipid droplets in adipocytes and hepatocytes [7]. Thus, autophagy activation in the RPE may improve the clearance of insoluble lipofuscin while promoting degradation of the daily lipid load faced by RPE during OS phagocytosis and lipoprotein particle uptake. Efficient degradation of ingested lipids may, in turn, decrease secretion of drusen-inducing lipoprotein particles. Further, breakdown of fatty acids may induce ketone body (KB) production by the RPE. In turn, KB secretion by the RPE, which is almost exclusively apically directed towards photoreceptors, has been shown to provide photoreceptors with an alternate fuel source and may promote photoreceptor survival under stress [8, 9]. Thus, autophagy activation has multiple theoretical mechanisms for alleviating AMD phenotypes [10, 11].

While hundreds of small molecule and protein targets for autophagy induction have been published [12–34], we and others have previously shown that small molecule inducers of autophagy in one cell type often do not induce autophagy in other cell types [13]. Almost none of the hits from prior autophagy inducer screens have been tested for efficacy and toxicity in RPE. Further, most of the autophagy inducers tested in RPE directly inhibit mTOR or its immediate upstream kinases. Strong mTOR inhibition may disrupt RPE phagocytosis and has failed in a randomized-controlled trial for advanced dry AMD [35–37]. These data suggest methods other than direct mTOR inhibition are needed for exploring the therapeutic potential of autophagy in the RPE.

Non-primate models that replicate the features of dry macular degeneration are not available. While some genetic mouse models simulate some features of drusen, none closely recapitulate human drusen morphology and composition [38–40]. Additionally, the structure and composition of lipoprotein particles that underpin human drusen development are markedly different in mice [41]. To complement shortcomings in mouse models, primary human RPE culture models of lipoprotein secretion and drusen formation have been established [40, 42, 43].

Here, we screened small molecule activators of autophagy in primary human RPE culture that are not known to directly target mTOR or its proximal upstream kinases. One of these activators, an FDA-approved anti-helminthic called flubendazole (FLBZ), promotes degradation rather than secretion of ingested lipids, leading to production of photoreceptor-protective KBs while decreasing secretion of drusen-forming apolipoprotein. FLBZ also decreases the burden of lipofuscin accumulation while alleviating lipofuscin-induced senescence and tight-junction disruption. *In toto*, these induced changes are predicted to alleviate pathology in dry AMD.

## RESULTS

### Identifying autophagy inducers in RPE

We selected over 30 putative autophagy inducers that are not known to directly inhibit mTOR or its immediate upstream kinases and tested these inducers in a primary human fetal RPE (hfRPE) culture system [44]. Nearly all of the autophagy inducers chosen were FDA-approved compounds or have a clearly defined protein target under pharmacologic development. Previous literature suggested all compounds should induce autophagy at a low μM concentration, with the exception of fenofibrate and metformin, which have high serum concentrations at clinically relevant doses. Compounds with highly toxic mechanisms (e.g. alkylating agents) were excluded from testing, and within a pharmacologic class, no more than two compounds were tested (Table 1).

**Table 1:** Putative autophagy inducers selected for study in primary human RPE cultures.

**Name**	**Abbreviation**	**Concentration tested in this study**	**Citation**
**GSK 1059615**	**GSK**	**10 μM**	[46]
**Torin1**	**Torin**	**1 μM**	[47]
**D4476**		**10 μM**	[20, 48]
**Flubendazole**	**FLBZ**	**12 μM**	[12]
**Amiodarone**		**10 μM**	[49]
**GW7647**		**1 μM**	[14]
**JNJ-47965567**	**JNJ**	**10 μM**	[33]
2-Acetyl-5-tetrahydroxybutyl Imidazole	THI	10 μM	[21]
Ac-Calpastatin		10 μM	[50]
AZ-10606120		10 μM	[33]
Bortezomib		50 nM	[51]
BRD5631		10 μM	[30]
Carbamazepine		10 μM	[52]
Clonidine		10 μM	[52]
Entinostat	MS-275	10 μM	[53, 54]
Erlotinib		10 μM	[55]
Fasudil	HA-1077	10 μM	[56]
Fenofibrate		200 μM	[57]
Fluphenazine		10 μM	[58]
K604		10 μM	[27]
Loperamide		4 μM	[23]
Metformin		1 mM	[28]
ML246	Metarrestin	10 μM	[19]
Mocetinostat		10 μM	[53, 54]
Nilotinib		10 μM	[59]
Nilvadipine		10 μM	[60]
Oxaprozin		10 μM	[31]
Rilmenidine		10 μM	[61]
Saroglitazar		10 μM	[62, 63]
Sertraline		10 μM	[64]
Spermidine		10 μM	[26]
Trifluoperazine		1 μM	[23]
(±)-Verapamil		10 μM	[28]

Direct mTOR inhibitors that induced autophagy in primary human fetal RPE cultures are highlighted in red. Compounds that do not directly inhibit mTOR but did induce autophagy in RPE cultures by both analysis of LC3 lipidation and LC3 puncta formation are highlighted in orange. Compounds that induced autophagy in RPE culture by only LC3 lipidation but not puncta formation are highlighted in green. All other compounds failed to induce LC3 lipidation in RPE cultures.

**Figure 1:**
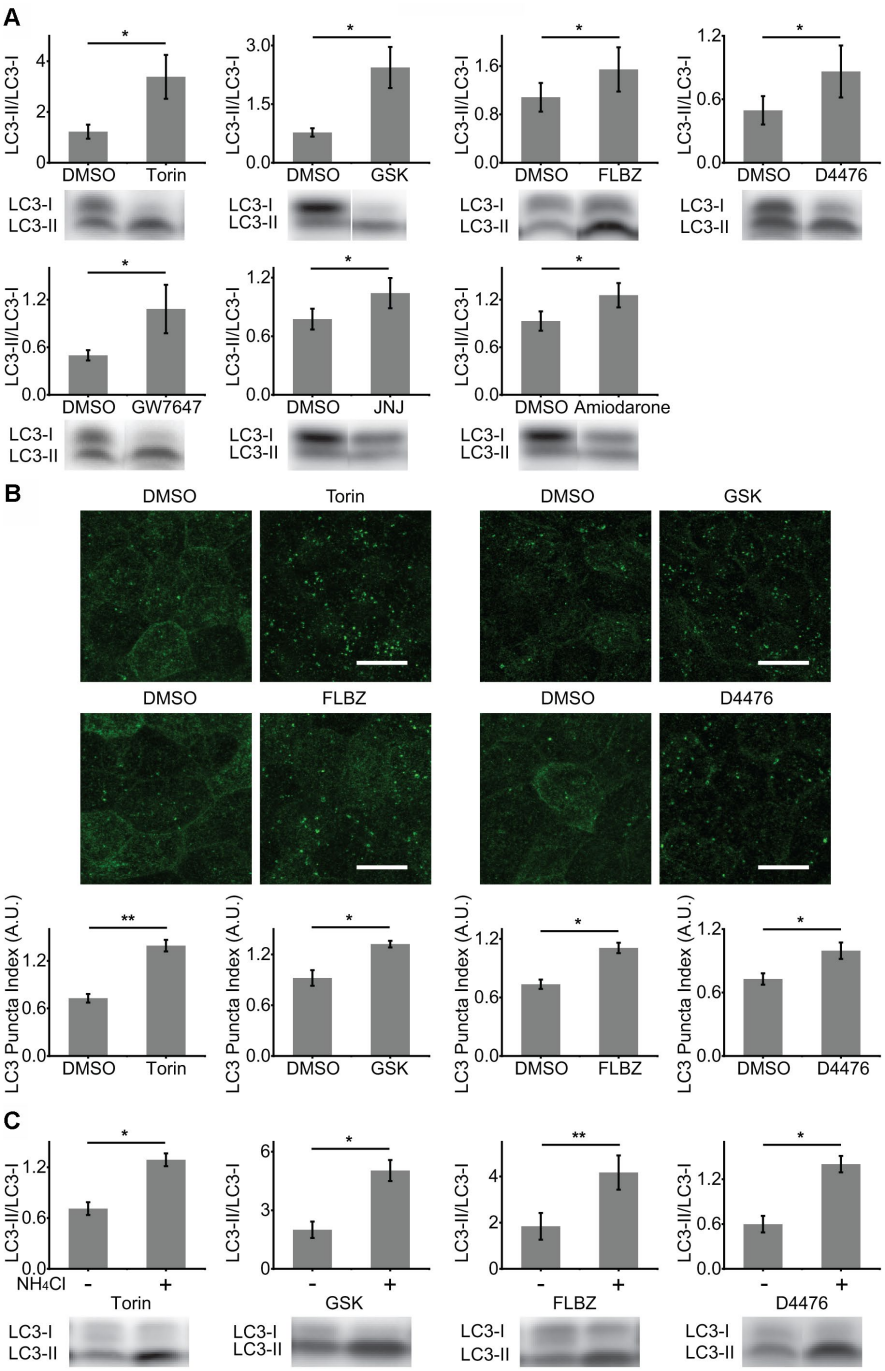
**Identification of autophagy inducers in primary hfRPE culture.** (**A**) Induction of autophagy by analysis of LC3 lipidation (LC3-II/LC3-I ratio). Cultures were exposed to compounds or vehicle (DMSO) for 24 hours. Torin n=8, GSK n=10, FLBZ n=10, D4476 n=8, GW7647 n=11, JNJ n=10, Amiodarone n=8. (**B**) Induction of autophagy by analysis of LC3 puncta formation (LC3 staining in green) using LC3 Puncta Index described previously [44]. Scale bar: 10 μm. Torin n=7, GSK n=3, FLBZ n=4, D4476 n=7. (**C**) Autophagy flux assays. After application of inducers or vehicle (DMSO) for 22.5 hours, 25mM of NH_4_Cl, a lysosomal alkalizing agent, or H_2_O were added for a final 1.5 hours to inhibit autophagy flux. Resulting increases in the LC3-II/LC3-I ratio indicate that the compound induces autophagy flux. Torin n=6, GSK n=3, FLBZ n=6, D4476 n=5. Uncropped blots for Figure 1 in Supplementary Figure 3. *p < 0.05, **p < 0.01.

To test for autophagy induction, we measured lipidation of the core autophagy protein LC3 by mobility shift on Western blots 24 hours after each compound was added to hfRPE cultures [45]. We first confirmed that primary RPE cultures upregulate autophagy in response to classical inducers, including mTOR inhibition using the mTOR-specific inhibitor Torin1 (Torin, Figure 1A) and the upstream pan-phosphoinositide-3-kinase and mTOR dual inhibitor GSK1059615 (GSK, Figure 1A) as well as amino acid/serum starvation (Supplementary Figure 1). Of the more than 30 putative autophagy inducers tested, only five produced an increased ratio of LC3-II/LC3-I, consistent with increased autophagy (Figure 1A).

Autophagy induction was confirmed by assaying for the formation of LC3-positive puncta (autophagosomes) by immunocytochemistry [45]. Puncta formation was quantified in automated/unbiased fashion using a previously published customized macro in the Fiji/ImageJ platform [44]. Besides Torin and GSK, only two compounds, D4476, a casein kinase 1 inhibitor, and FLBZ, an FDA-approved anti-helminthic, also induced autophagy by this second assay (Figure 1B).

Increases in both LC3 lipidation and autophagosome formation could result from downstream blockade of autophagy at the lysosome. To confirm that our hits were genuine inducers of RPE autophagy flux, we disrupted the last step of autophagy flux, lysosomal degradation, through alkalinization of the lysosome with ammonium chloride, a well-accepted method for confirming autophagy flux [45]. As expected for elevated autophagy flux rather than downstream autophagy blockade, levels of lipidated LC3 rose for all small molecule autophagy inducers (Figure 1C) and for amino acid/serum deprivation (Supplementary Figure 1). Autophagy flux can also be demonstrated by the appearance of puncta containing phosphorylated ATG16L1, which decorates newly formed autophagosomes but, unlike LC3, rapidly dissociates as the autophagosome matures [65]. Thus, the number of phospho-ATG16L1 puncta does not change with downstream blockade of autophagy. Phospho-ATG16L1 immunofluorescence confirms increased autophagy flux for both mTOR-independent autophagy inducers, FLBZ and D4476, while also demonstrating an expected decreased autophagy flux for the lysosomal poison chloroquine (Supplementary Figure 2).

### Safety of autophagy inducers

**Figure 2:**
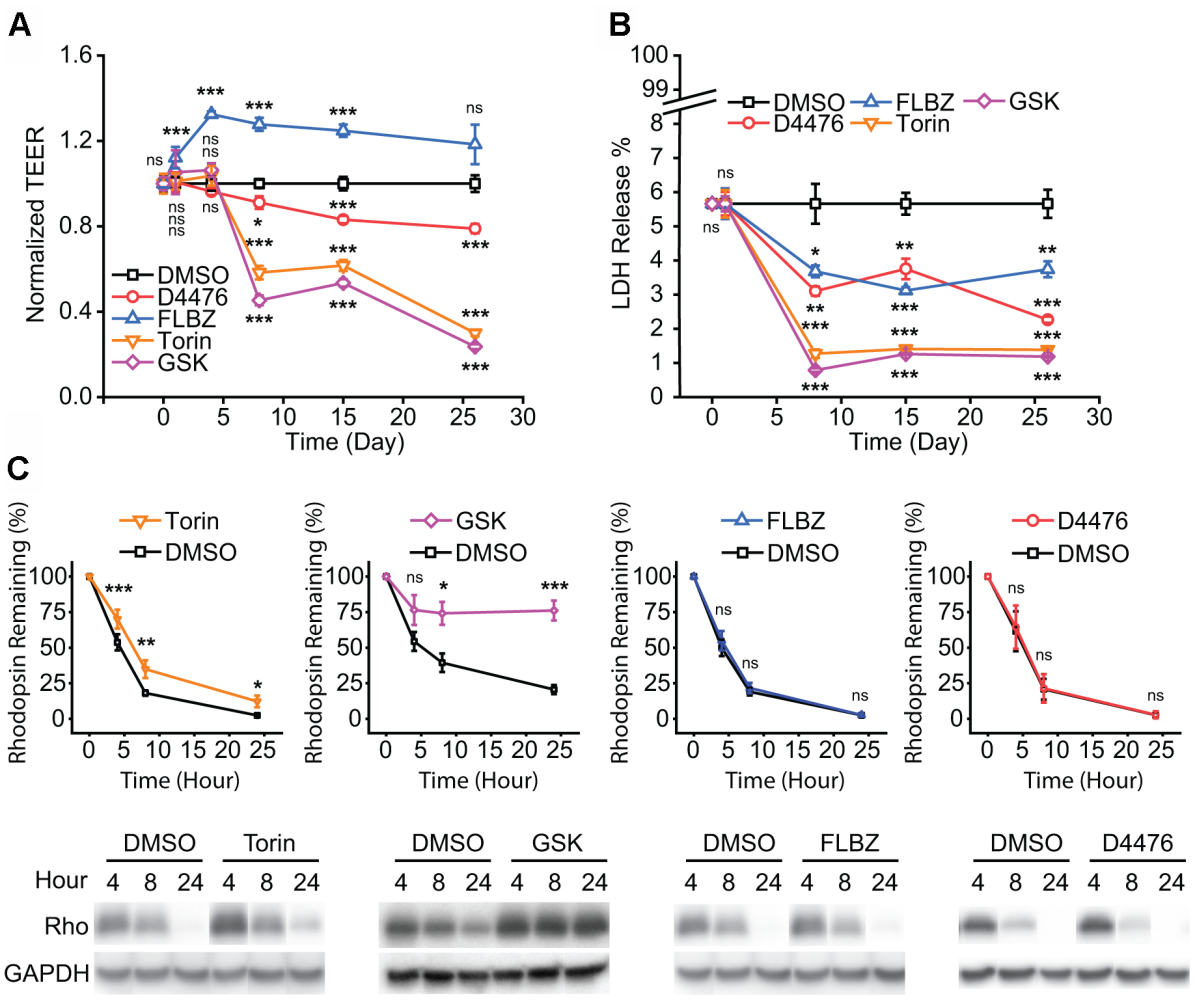
**Safety of confirmed autophagy inducers in primary hfRPE culture.** (**A**) Tight-junction integrity, as measured by trans-epithelial electrical resistance (TEER), is a general marker of RPE health. Drug or vehicle (DMSO) replaced daily with measurement just before drug replacement. FLBZ shows enhanced TEER while all others, especially mTOR inhibitors Torin and GSK, demonstrate progressively lower tight-junction integrity. n=6. (**B**) Cytotoxicity as measured by percent of total possible LDH release into the apical supernate. Drug or vehicle (DMSO) replaced daily with supernate collected just before drug replacement. All compounds demonstrated slightly lower cytotoxicity than DMSO control. Note scale break on Y-axis, indicating all conditions, including vehicle, demonstrated minimal LDH release. n=6. (**C**) Outer segment (OS) phagocytosis efficiency, as measured by disappearance of rhodopsin, the primary protein in OS. Purified OS are fed to RPE cultures and Western blotting for rhodopsin (Rho) indicates undigested OS remaining, as elaborated in Methods. Direct mTOR inhibition (Torin, GSK) reduces phagocytosis efficiency, whereas D4476 and FLBZ have no effect on phagocytosis. Representative blot below each graph, with GAPDH bands demonstrating equal cell mass across Transwells used for the phagocytosis assays. Torin n=12, GSK n=4, FLBZ n=9, D4476 n=3. Uncropped blots for Figure 2 in Supplementary Figure 6. ns p > 0.05, *p < 0.05, **p < 0.01, ***p < 0.001.

To evaluate the safety of D4476 and FLBZ, we assessed trans-epithelial electrical resistance (TEER), a measure of tight-junction integrity, in our RPE cultures after prolonged exposure to each compound. As well-formed tight-junctions require myriad cell processes to be optimally coordinated, assessing TEER provides an easily measurable, ongoing, and non-invasive marker for general RPE cell health [66]. While both mTOR inhibitors, Torin and GSK, dramatically reduced TEER, D4476 had a more modest impact and FLBZ had no negative effect on TEER (Figure 2A). Reduced RPE pigmentation is associated with increased susceptibility to oxidative insults [67], and in contrast to cultures treated with repeated doses of FLBZ or vehicle for at least 20 days, Torin reduced RPE pigmentation (Supplementary Figure 4). Lactate dehydrogenase (LDH) release, a combined marker of necrotic and late-stage apoptotic cell death [68, 69], was reduced compared to vehicle for all four verified RPE autophagy inducers (Figure 2B). Exposure of RPE cultures to the oxidant tert-butyl hydroperoxide confirmed the ability of the assay to detect cell death (Supplementary Figure 5).

Daily OS phagocytic uptake and degradation is a core RPE function necessary for retinal function [70]. A previous study suggested that induction of autophagy in the RPE leads to impaired OS degradation after initial phagocytic internalization since autophagy and RPE phagocytosis share overlapping protein machinery [29]. While we found that autophagy induction with mTOR inhibitors did indeed impair breakdown of OS, D4476 and FLBZ had no effect on OS degradative capacity (Figure 2C).

### Effects of autophagy inducers on the balance between lipid degradation and lipid secretion

**Figure 3:**
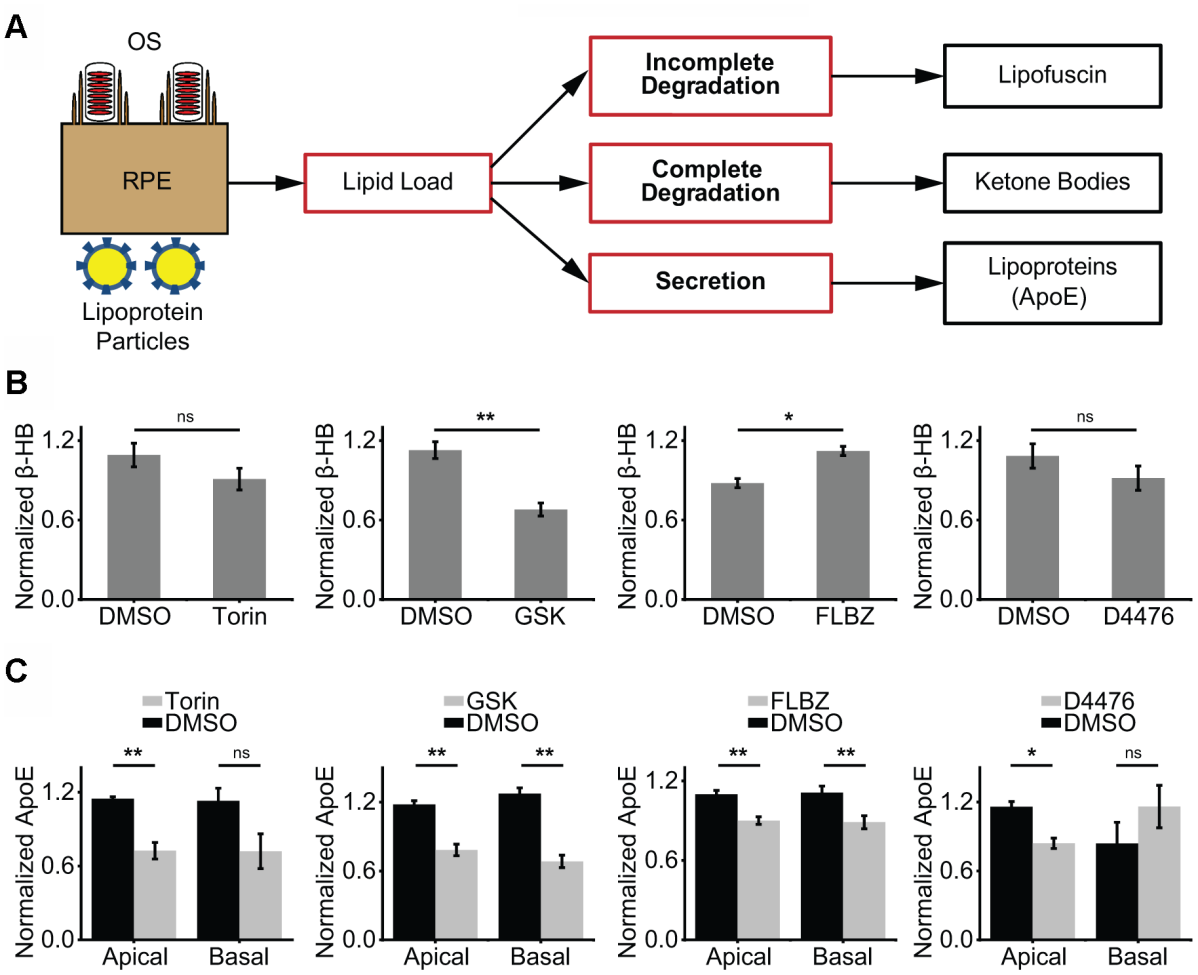
**Impact of confirmed autophagy inducers on RPE lipid metabolism.** (**A**) Proposed model of RPE lipid handling. Lipid-rich shed OS are phagocytosed from the apical side and lipoprotein complexes are consumed from the basolateral side daily. Incomplete lipid degradation contributes to lipofuscin accumulation. With the remaining lipid load, we postulate that the RPE daily determines the balance between complete lipid degradation, as assessed by KB production, and secretion of lipid via lipoprotein particles, as assessed by apoE secretion. (**B**) KB production (as assessed by β-hydroxybutyrate, β-HB) in the presence of vehicle (DMSO) or confirmed autophagy inducers for 24 hours. β-HB is secreted almost exclusively into the apical supernate. Only FLBZ increased lipid degradation. Torin n=4, GSK n=7, FLBZ n=6, D4476 n=4. (**C**) Apolipoprotein secretion (as assessed by apoE) in the presence of vehicle (DMSO) or confirmed autophagy inducers for 24 hours. Both apical and basolateral media contain apoE. While increasing lipid degradation in (**B**), FLBZ also decreases secretion of drusen-promoting apolipoprotein. Apical: Torin n=4, GSK n=5, FLBZ n=11, D4476 n=4. Basal: Torin n=4, GSK n=5, FLBZ n=9, D4476 n=3. ns p > 0.05, *p < 0.05, **p < 0.01.

The RPE handles an enormous lipid burden on a daily basis, including OS ingestion from its apical side and lipoprotein particle absorption from its basolateral side. Rather than storing excess lipid, the RPE may choose to degrade or secrete surplus lipids (Figure 3A). A marker of lipid degradation is the production of KBs, which are secreted by the RPE apically and serve as an energy source for photoreceptors [8, 9]. A marker of lipid secretion is the production of the apolipoprotein, apoE, which is a major component of drusen [71]. We hypothesized that autophagy inducers may promote ketogenesis through degradation of ingested lipids, reducing the stimulus for secretion of drusen-promoting apoE. In our primary RPE cultures, only FLBZ both increased production of the major KB, β-hydroxybutyrate (β-HB), and reduced the apical and basolateral secretion of apoE, suggesting that FLBZ-mediated autophagy induction changes the lipid-handling profile of RPE away from drusen promotion (Figure 3B, 3C).

### Effects of autophagy inducers on lipofuscin-like accumulation

**Figure 4:**
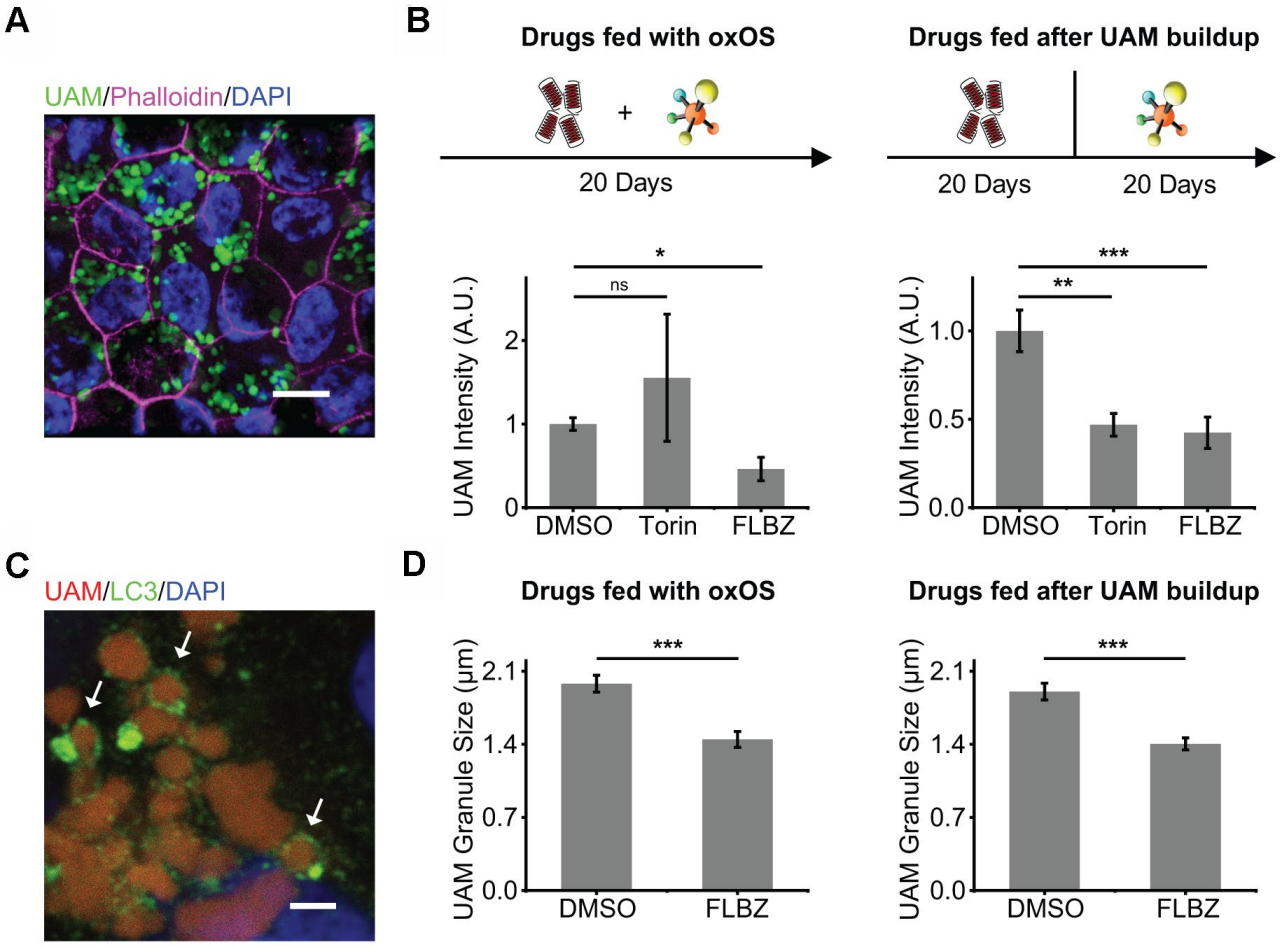
**Autophagy inducer FLBZ reduces accumulation of lipofuscin-like material.** (**A**) Lipofuscin-like UAM accumulates in RPE after repeated feedings of photo-oxidized outer segments (oxOS). UAM granules (green). DAPI (blue). Phalloidin stain of F-actin outlining cell borders (pink). Scale bar: 10 μm. (**B**) Effects of FLBZ or Torin on UAM accumulation (left) and elimination (right). FLBZ or Torin is fed together with oxOS daily for 20 days in a month (left, n=5) or fed daily for 20 days in a month after completion of oxOS feedings to stimulate UAM accumulation (right, n=7). Unlike Torin, FLBZ both reduces UAM accumulation and increases UAM elimination. DMSO as vehicle control. UAM normalized to DMSO condition. (**C**) LC3 colocalization to UAM granules in the human RPE cell line, ARPE-19, treated with FLBZ. UAM (red). LC3 (green). DAPI (blue). Arrows indicate LC3 puncta surrounding a lipofuscin granule. Scale bar: 2 μm. (**D**) Effects of FLBZ on UAM granule size. Compared to vehicle (DMSO), FLBZ decreases UAM granule size both during oxOS feedings (left) and after UAM buildup has already occurred (right). n=40. ns p > 0.05, *p < 0.05, **p < 0.01, ***p < 0.001.

We have developed and extensively characterized a model of lipofuscin-like material accretion through repeated feeding of photo-oxidized OS (oxOS) to primary hfRPE culture [72]. Twenty-plus oxOS feedings over the course of a month results in a significant and stable autofluorescent granule burden (Figure 4A). With time, these granules, which we term undigestible autofluorescent material (UAM), resemble the size and emission spectrum of lipofuscin. Like lipofuscin, UAM stain with Nile Red, a marker of neutral lipids, and frequently combine with melanosomes to form melanolipofuscin granules [72].

The concurrent feeding of oxOS and FLBZ at 12μM over the course of a month resulted in significantly less UAM accumulation compared to feedings of oxOS plus vehicle (Figure 4B). Since FLBZ has no effects on phagocytosis efficiency (Figure 2C), this reduction in UAM accumulation was not due to less uptake of oxOS in the FLBZ group. We also saw LC3 colocalization with UAM granules, supporting a role of autophagy in clearing UAM (Figure 4C). Remaining UAM granules in FLBZ-treated cultures were smaller than untreated cultures (Figure 4D). In contrast, treatment with Torin at 1μM over the course of a month resulted in more UAM accumulation (Figure 4B), possibly due to inhibitory effects of Torin on phagocytic degradation (Figure 2C).

To determine whether FLBZ could reduce UAM after it had accumulated, we fed our cultures with oxOS twenty times over the course of a month, then extensively washed off OS from the cultures, and subsequently treated with twenty repeated drug feedings over the course of an additional month. FLBZ led to significantly lower levels of UAM and smaller granule size, confirming that UAM is compactable and/or removable even after its accrual in the RPE (Figure 4B, 4D).

**Figure 5:**
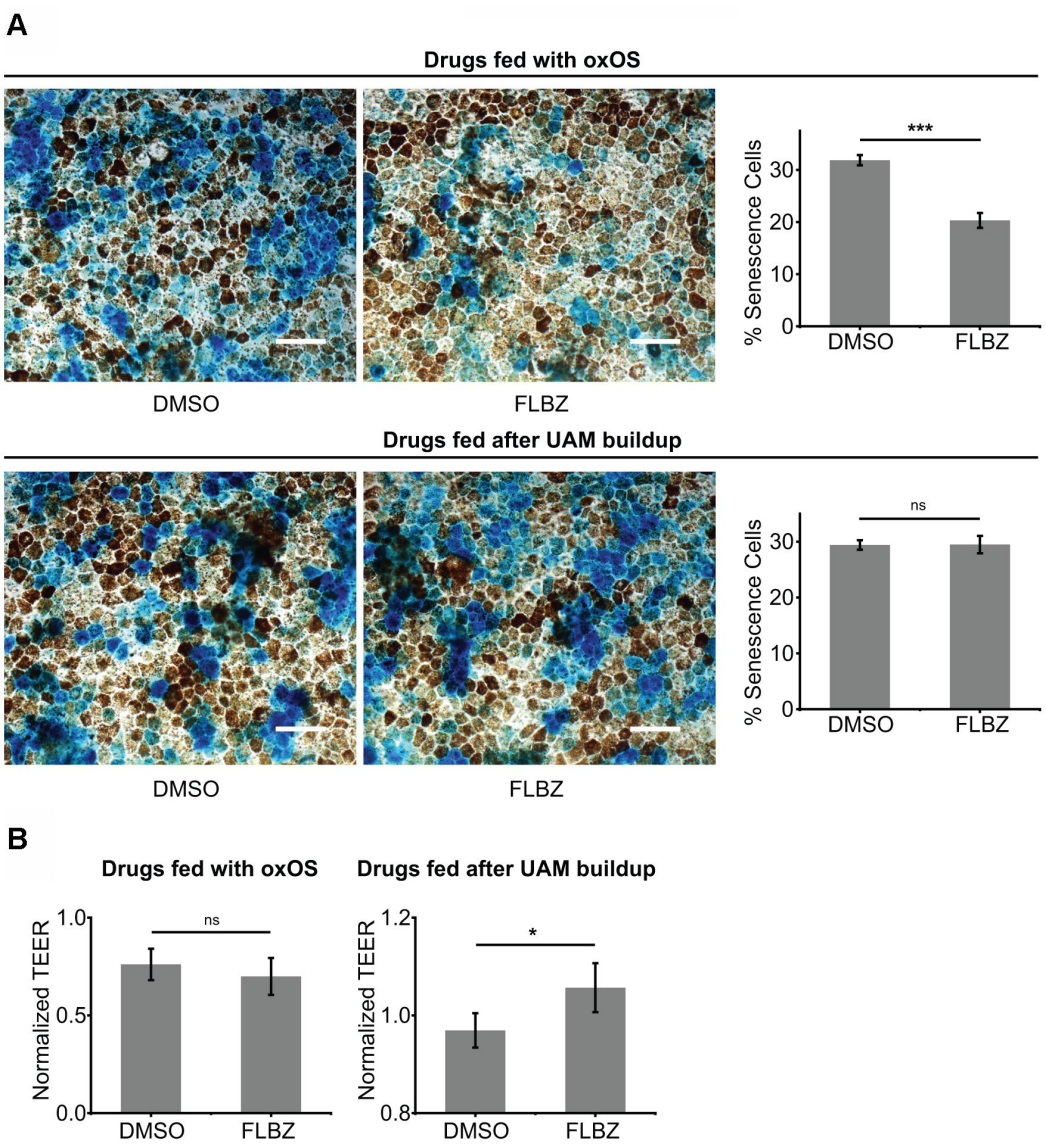
**FLBZ alleviates UAM-induced senescence and tight-junction disruption.** (**A**) (Top) FLBZ reduces senescence when fed concurrently with oxOS during UAM accumulation. (Bottom) FLBZ is unable to reverse established senescence induced by already accumulated UAM. FLBZ is fed daily together with oxOS for 20 days in a month (top) or after one month of 20 oxOS feedings to induce UAM buildup (bottom). DMSO as vehicle control. Senescence measured by β-galactosidase activity (blue). Scale bar: 50 μm. n=6. (**B**) While FLBZ does not reduce senescence when added to culture after UAM accumulation has already occurred, it does improve RPE cell health, as assessed by tight-junction integrity (TEER measured after 20 FLBZ feedings) (right). Left graph n=6, right graph n=12. ns p > 0.05, *p < 0.05, ***p < 0.001.

We and others have shown that cultures with a high UAM burden demonstrate significant senescence [72, 73], which in the RPE can contribute to the para-inflammatory state characteristic of AMD [74]. Cofeeding cultures with oxOS and FLBZ resulted in less senescence compared to feeding of oxOS plus vehicle (Figure 5A). Interestingly, when we treated cultures with FLBZ after UAM had already accumulated, cell senescence was not decreased, suggesting that established RPE senescence may be difficult to reverse (Figure 5A). Nevertheless, FLBZ still improved cell health, as assessed by tight-junction integrity, in cultures with already established UAM (Figure 5B).

## DISCUSSION

Dry AMD may be a disease of perturbed lipid homeostasis, characterized by extracellular deposition of lipid-rich drusen. The accumulation of lipid-rich intracellular lipofuscin may also be linked to AMD and Stargardt macular dystrophy [75, 76]. Efficient degradation of ingested lipid by the RPE produces KBs that are secreted and then utilized by photoreceptors for metabolism. RPE ketogenesis may promote survival of metabolically-stressed photoreceptors deprived of their primary energy source, glucose [8, 9]. For all these reasons, improving RPE lipid handling is an attractive therapeutic approach for the treatment of dry AMD.

Autophagy induction, which promotes lipid degradation and turnover of otherwise undigestible cellular constituents, has the potential to favorably impact RPE lipid homeostasis. However, in this study, we show that mTOR inhibition, the most common pathway for inducing autophagy, disrupts multiple RPE-specific functions. Furthermore, the mTOR inhibitor, sirolimus, failed to demonstrate therapeutic benefit in a randomized controlled trial of advanced dry AMD [37]. While researchers have pharmacologically induced autophagy in the RPE in a variety of cell and animal culture models [77–79], no explicit attempt has been made to identify lead compounds that do not directly inhibit mTOR or its immediate upstream kinases. We therefore sought to identify non-toxic autophagy inducers in primary human RPE independent of direct mTOR inhibition.

Of more than 30 putative autophagy inducers we tested, only two, D4476 and FLBZ, reliably induced autophagy in our primary human RPE cultures. Our results support prior assertions that autophagy inducers are often cell specific [13].

In evaluating autophagy inducers in the RPE, we found that mTOR inhibition disrupted RPE-specific functions, including tight-junction integrity, OS phagocytosis, and pigmentation. The role of mTOR in RPE health is multifaceted. On the one hand, over-activation of mTOR leads to RPE dedifferentiation through an epithelial-to-mesenchymal transition [80]. mTOR activation is also associated with RPE senescence [81] and less efficient phagocytosis of OS [82]. On the other hand, timely activation of mTOR and upstream kinases during OS ingestion is integral to coordinated phagocytosis [35, 36, 83], and activation of mTOR may be important for RPE survival under stress [84]. Given the deleterious effects of mTOR inhibition on RPE function in our primary culture system, we propose that the RPE requires a careful and time-dependent balance between mTOR activating and deactivating signals. Direct or immediate upstream modulation of mTOR may not, therefore, be therapeutically tractable.

In our model of RPE lipid handling (Figure 3A), the RPE ingests OS and lipoprotein particles as part of a daily lipid challenge. If OS are incompletely degraded, lipofuscin develops. Lipid that is fully degraded in the lysosome transits the endoplasmic reticulum and is packaged as lipid droplets. The lipid droplets that form after an RPE lipid challenge, however, dissipate quickly [85], in contrast to the longer-lived lipid droplets of adipocytes or hepatocytes. Further, the accumulation of bloated lipid vacuoles, a feature of many age-related diseases including atherosclerosis [86], cardiomyopathy [87], liver disease [86], and neurodegeneration [88, 89], is not a feature of RPE degeneration in macular degeneration. Thus, the RPE’s large daily lipid load is actively degraded or secreted rather than stored long-term. Tipping the balance towards degradation may provide photoreceptors with KBs, an alternative fuel source, and decrease the amount of secreted lipoprotein that contributes to drusen formation. We found FLBZ altered the balance between degradation vs. secretion of lipid in our primary human RPE culture. By reducing lipid secretion, FLBZ has the potential to work synergistically in dry AMD with pharmacologic programs aimed at clearing already deposited lipid/drusen in Bruch’s membrane [90, 91].

Current pharmaceutical approaches to lipofuscin reduction have focused on disrupting the retina’s visual cycle, which produces the retinoids that contribute to lipofuscin accumulation. However, all visual cycle modulators have failed to date in human clinical trials, likely because the visual cycle is so integral to visual function [92]. Avoiding the disadvantages of visual cycle modulation, we found that autophagy induction both prevented and reduced the accumulation of lipofuscin-like material (i.e. UAM [72]) in primary RPE culture.

Autophagy may clear lipofuscin both by wholesale engulfment of granules in a process akin to autophagic engulfment of lysosomes [93] and by the natural upregulation of lysosomal capacity that accompanies autophagy induction [94, 95]. Alternatively, components of lipofuscin are known to trigger accumulation of cholesterol in the lysosome and reduce cholesterol concentration in cell membranes [96]. As phagosome transport to the lysosome depends on cholesterol-rich lipid rafts [97], alterations in cholesterol composition of cell membranes retards phagosome maturation, which may lead to further accumulation of lipofuscin. There is also evidence that lipofuscin-mediated accumulation of cholesterol in the lysosome indirectly triggers altered microtubule dynamics which, in turn, slows phagosome and autophagosome maturation [98], leading to further lipofuscin accumulation. Induction of autophagy may directly or indirectly alter the cholesterol dyshomeostasis triggered by lipofuscin, leading to improved degradation of OS components and decreased lipofuscin accumulation.

Under conditions where FLBZ prevented UAM accumulation, the drug also reduced senescence associated with UAM. Reduced senescence may decrease AMD-associated inflammation and neovascularization [99, 100], and drugs that specifically eliminate senescent cells are in early stage clinical development for macular degeneration [101, 102]. Consistent with reports on the difficulty of reversing senescence [103], FLBZ did not reduce senescence in cultures where UAM had already accumulated. Nevertheless, delaying FLBZ treatment until after UAM had fully accumulated still reduced UAM burden, compacted remaining UAM granules, and modestly improved cell health, as assessed by tight-junction integrity. We have previously shown that UAM granules slowly compact over 12 months in culture; the acceleration of this compaction process by autophagy may diminish reactivity and toxicity of lipofuscin-like granules [72]. Consistent with this hypothesis, lipofuscin granules in AMD are larger than those in normal age-related controls [104].

The mechanism of FLBZ’s induction of autophagy is not fully understood. FLBZ is an agonist of ATG4, a cysteine protease necessary for cleaving the core autophagy protein LC3 into its active form [105]. Additionally, FLBZ appears to trigger depolymerization of dynamic microtubules (MT), while stabilizing acetylated MTs, a property unique among a range of other microtubule depolymerizing agents [12]. MT acetylation-triggered activation of the stress kinase JNK1 leads to Bcl-2 phosphorylation which, in turn, releases the upstream autophagy inducer Beclin1 from its inhibitory complex with Bcl-2 [12]. Interestingly, the single largest genetic risk factor for AMD, the HTRA1 locus, codes for a serine protease that, like FLBZ, has significant effects on MT stability [106]. Whether FLBZ’s effects on autophagy and AMD histologic phenotypes described here depend on HTRA1 allele status is an area for further research.

Our study utilized primary human fetal RPE to model aspects of AMD. There are advantages and limitations with this model. Any culture model lacks the complete retinal ecosystem that contextualizes RPE function in animal models [107], and there is clear evidence for the involvement of the choroid, Bruch’s, RPE, and photoreceptors in AMD pathogenesis. On the other hand, no AMD mouse model truly recapitulates drusen formation [3]. Certain models show Bruch’s membrane thickening with potential cholesterol deposition but without the lipoprotein particle composition that dominates human drusen [3, 108]. Further, the entire lipoprotein system, fundamental for drusen formation in humans, is markedly different between mice and humans [41].

The fetal origin of our human RPE cultures is also conceptually at odds with modeling a disease whose overwhelming principal risk factor is age. Indeed, while lipofuscin formation and oxidative stress may play a role in AMD, human fetal RPE cultures demonstrate a robust resistance to lipofuscin accumulation [72] and oxidative stress, tolerating doses of the oxidant tert-butyl hydroperoxide that typically trigger significant damage and death in other RPE culture models [109–112]. On the other hand, our hfRPE cultures demonstrate deposition of apoE and hydroxyapatite drusen-like deposits in the Transwell, characteristic of AMD [113] (data not shown). Further, in contrast to primary adult human RPE cultures, hfRPE expresses the drusen-promoting apolipoprotein, apoE, at a high level that closely approximates apoE expression in human RPE *in vivo* (data not shown). To ensure our hfRPE cultures mimic *in vivo* RPE to the maximum extent possible, we grow hfRPE exclusively on Transwells for, on average, at least 3 months; only cultures with high TEER (usually > 500 Ω*cm^2^), high pigmentation, and uniformly cobblestone morphology are utilized for all experiments. We have previously shown, under these conditions, that the RPE maintains numerous characteristics of *in vivo* RPE [44].

In summary, we demonstrate that activation of autophagy through mechanisms that do not involve direct mTOR inhibition can mitigate the processes associated with extracellular drusen formation and intracellular lipofuscin accumulation without causing RPE toxicity. As drusen and lipofuscin are pathologic hallmarks of dry AMD, FLBZ is a promising candidate drug for this disease.

## MATERIALS AND METHODS

### Primary hfRPE and ARPE-19 culture

Human fetal eyes were obtained from Advanced Bioscience Resources (ABR, Alameda, California) and cultured according to previously published methods [44]. hfRPE was plated on Transwells at passage 1, and experiments were performed on hfRPE that was in culture, on average, for three months or more. All Transwell cultures demonstrate high pigmentation, cobblestone morphology, absence of any intertwined fibroblastic patches, and TEER of at least 375 Ω*cm^2^. Step-by-step directions for our hfRPE culture are available at https://medicine.umich.edu/sites/default/files/content/downloads/Human_RPE_Culture_Protocol.pdf. ARPE-19 cells were a gift from the Hjelmeland laboratory [114] and were cultured at passage 19 as previously described [72].

### Assaying for autophagy

Cell cultures were exposed to each putative autophagy inducer at a concentration indicated in Table 1 for 24 hours, lysed with 36μL of SDS sample buffer with β-mercaptoethanol, with 15μg of lysate loaded on a 4-15% gradient gel followed by transfer to a PVDF membrane. Blots were incubated with 1:1000 of LC3A/B antibody (Cell Signaling Technology, #4108s) overnight. Quantifying LC3-II/LC3-I ratio is a well-accepted method for determining autophagy induction [45], and this ratio was quantified in a non-saturated, linear range using the Azure c500 Imaging System (Azure Biosystems, Dublin, CA, USA) and a combination of AzureSpot and ImageJ software. The data in Figure 1A are not normalized, whereas the data in Figure 1B, 1C are normalized to the average combined value of the vehicle and drug groups within each experimental repeat. The extreme pigmentation of human fetal cultures, combined with the destruction of the LC3 epitope with melanin bleaching protocols, makes detection of LC3 by immunofluorescence in hfRPE difficult. We previously extensively tested a range of LC3 staining and puncta quantification protocols specifically adapted to pigmented hfRPE culture, and those were employed for this study [44]. At least five fields of view were randomly chosen, imaged with a Leica SP5 confocal microscope, and averaged together for each experimental replicate. Vendor and product number for autophagy inducers are listed in Supplementary Table 1. Phospho-ATG16L1 puncta immunofluorescence was performed on paraformaldehyde-fixed cells permeabilized with 50μg/mL of digitonin in PBS for 10 minutes at room temperature, followed by detergent-free conditions for the blocking, primary antibody, secondary antibody, and wash steps. The primary antibody (Abcam, #195242) was incubated in PBS + 1% BSA for 1 hour at room temperature at a dilution of 1:100. Autophagy flux in these experiments was blocked by incubating with 30 μM chloroquine (Sigma, #C6628) for 24 hours. Phospho-ATG16L1 puncta quantification was determined by counting puncta number for each condition and then normalizing this number to the average puncta number across all fields of view for both the control and treatment condition. This is reported as the “Puncta Index” in Supplementary Figure 2.

### TEER and cell death assays

TEER and cell death were measured as outlined previously [72]. For cell death assays, maximum possible LDH release per Transwell was measured immediately after 2μL of the final experimental supernate was taken. The Transwell and supernate was then treated with 0.2% Triton X-100 for 15 minutes at 37° C followed by collection of an additional 2μL of supernate. Each experimental LDH release value was first normalized to total LDH release from the vehicle condition at that timepoint. All TEER and LDH release measurements were then additionally normalized to the vehicle group at the zero hour timepoint. Step-by-step directions for our measurement of TEER in hfRPE cultures on Transwells are available at https://medicine.umich.edu/sites/default/files/content/downloads/Measuring_Transepithelial_Electrical_Resistance.pdf.

### Phagocytosis assays

Phagocytosis assays using bovine outer segments was performed using the “pulse-only” method described previously [44]. Briefly, in order to capture the total consumption of OS introduced to RPE in a single Transwell, the apical chamber of each Transwell is incubated with 50μL of media containing 4×10^6^ OS/mL and OS phagocytosis bridging ligands (recombinant human milk fat globule-EGF factor −8 (MFG-E8) (1.5μg/mL, Sino Biological, #10853-H08B) and purified Protein S (ProS) (4μg/mL, Enzyme Research Laboratories, #HPS)). In contrast to the pulse-chase method used in classical phagocytosis assays, OS were added (“pulse”) but not washed off (“chase”). At various times after OS “pulse”, we added 50μL T-PER lysis buffer (Thermo, #78510) plus complete protease inhibitor mini-tab (Thermo, #PIA32955) to the apical chamber. The collected lysate thus included both the RPE cell layer as well as the media above, containing the non-consumed OS. Once collected, the “supernatant + cell” lysate is subjected to SDS-PAGE and blotted with an N-terminally directed anti-rhodopsin antibody (1:5000 dilution, Encor BioTech, #MCA-B630). This assay provides an assessment of total OS consumption by staining for all non-consumed OS, whether those OS were in the media, bound on the RPE cell surface, or internalized but incompletely degraded. Rhodopsin values at the zero hour timepoint were used for normalization. Step-by-step directions for our OS isolation protocol are available at https://medicine.umich.edu/sites/default/files/content/downloads/Photoreceptor_Outer_Segment_Isolation.pdf.

### Measuring ApoE secretion and ketogenesis

After 24 hours of exposure to drugs, supernates were collected and subjected to western blotting for apoE detection and a fluorometric assay for β-HB detection (AAT Bioquest, #13831), as previously described [72]. Twelve microliters of apical and basolateral supernates for apoE were mixed with 4 μl of 4x sample buffer, run on SDS-PAGE, and blotted with antibody (Millipore, #AB947) at a dilution of 1:2000. ApoE secretion was assessed in the presence of our standard RPE culture media with 5% fetal bovine serum. Blotting of equivalent volumes of just RPE media with 5% fetal bovine serum demonstrates negligible amounts of apoE (data not shown), confirming that measured apoE in our experiments derive from RPE secretion and not bovine serum. ApoE and β-HB values were normalized to the average value of the vehicle and experimental group within each experimental repeat.

### Undigested autofluorescent material

UAM accumulation and quantification as well as assays on senescence were carried out as previously described [72]. UAM granule size was measured with Leica LAS X software using the length of the long axis of the granule ellipse.

### Statistical analysis

Means were compared using paired or unpaired two-tailed Student’s t-test, as appropriate. All error bars represent standard error of the mean unless otherwise specified. For apoE secretion experiments, there were non-balanced technical replicates between experiments and within treatment groups of a given experimental repeat. To ensure our normalization scheme did not lead to bias, we confirmed the magnitude and significance of our findings using mixed effects modeling in R [115]. All results from the mixed effects analysis were concordant with values and significance reported in this study.

### Role of funding source

Funders had no role in the conception, execution, or publication of this study. J.M.L.M. had full access to all data in this study and claims final responsibility for the decision to submit for publication.

## Supplementary Material

Supplementary Figures

Supplementary Table 1
