# Supplementary material for: Pharmacologic activation of autophagy without direct mTOR inhibition as a therapeutic strategy for treating dry macular degeneration
Source: Aging (Albany NY). 2021 Apr 19;13(8):10866–90. doi: 10.18632/aging.202974 (PMC8109132; doi:10.18632/aging.202974)
Supplement: Supplementary Figures [file aging-13-202974-s001.pdf]

SUPPLEMENTARY FIGURES

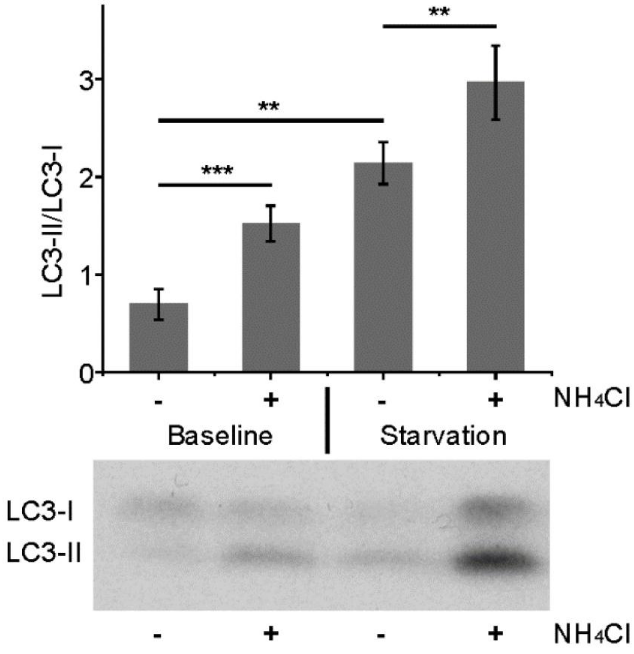

Supplementary Figure 1. Serum and amino acid starvation induce autophagy flux in hfRPE. Serum and amino acid starvation is induced by incubating hfRPE in Hank's Balanced Salt Solution for 6 hours, with normal media as control. The lysosomal alkalinizing agent NH4Cl (25 mM) was added 1.5 hours prior to harvest. In control wells, the increase in LC3-II/LC3-I ratio after blockade of autophagy flux by NH4Cl demonstrates that hfRPE has high baseline levels of constitutive autophagy. Amino acid and serum starvation with Hank's Balanced Salt Solution induces autophagy flux above baseline levels. n=6. ***p < 0.01, ***p < 0.001*.

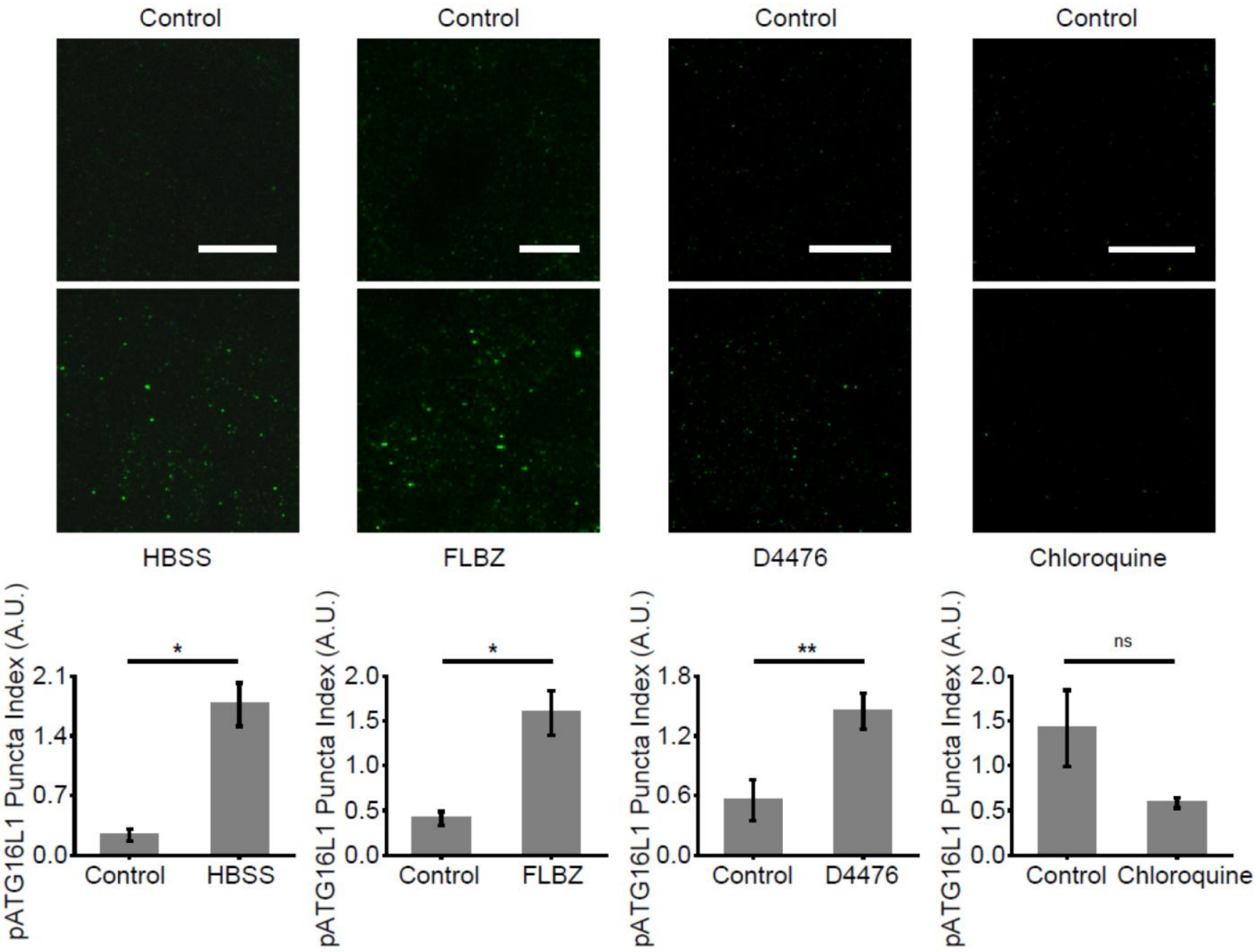

Supplementary Figure 2. Phospho-ATG16L1 puncta formation confirms that serum and amino acid starvation, FLBZ, and D4476 stimulate autophagy flux. Serum and amino acid starvation induced by incubating hfRPE in Hank's Balanced Salt Solution (HBSS) for 4 hours. Control cultures were incubated with normal media. FLBZ, D4476, and the lysosomal alkalinizing agent, chloroquine (30 µM), were incubated for 24 hours prior to immunostaining, with control wells containing the drug vehicle for FLBZ, D4476, or chloroquine. Phospho-ATG16L1 puncta staining demonstrates increased autophagy flux for serum and amino acid starvation, FLBZ, and D4476, and an expected decrease in flux with chloroquine treatment. Calculation of the puncta index is detailed in Methods. Scale bar: 10 µm. *ns $p > 0.05$, *$p < 0.05$, **$p < 0.01$*.

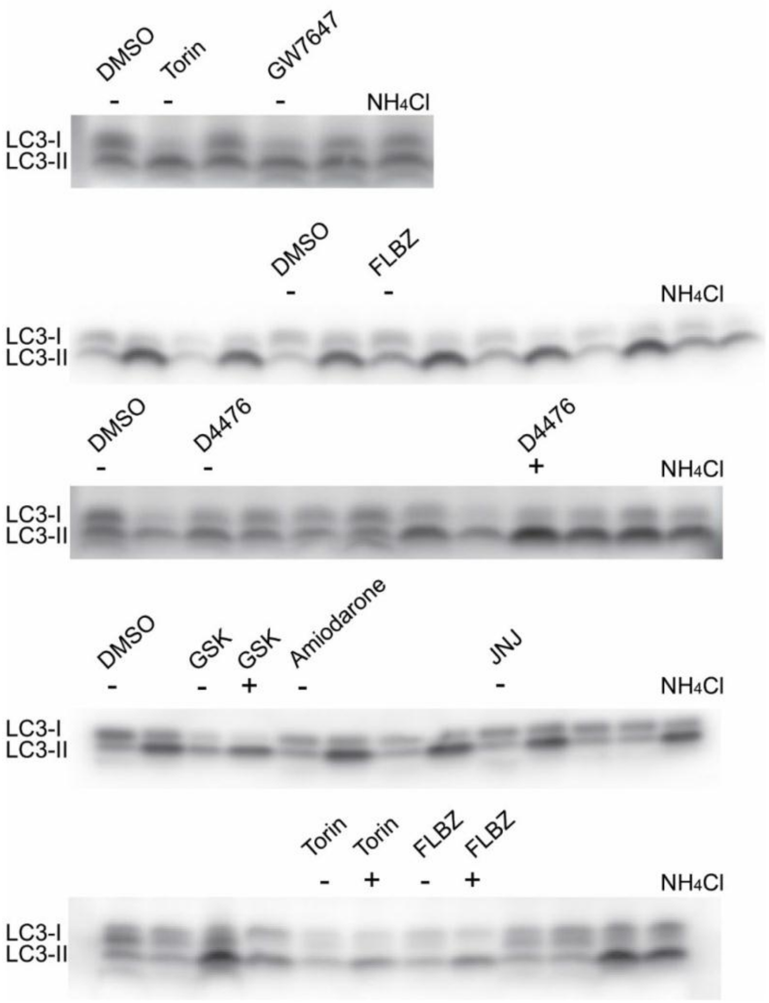

Supplementary Figure 3. Uncropped western blots for Figure 1. Symbols (+ or -) indicate presence or absence of NH_4Cl.

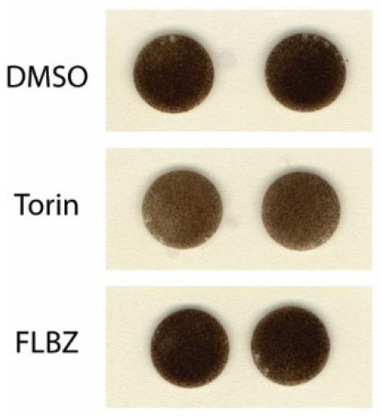

Supplementary Figure 4. mTor inhibitor Torin1 reduces hfRPE pigmentation. Whole mounted Transwells (in duplicate) are photographed. Daily feeding of oxOS and Torin together during UAM buildup resulted in reduced pigmentation (20 drug changes over 4 weeks). In contrast, an identical feeding protocol involving oxOS and FLBZ or oxOS and vehicle (DMSO) together during UAM buildup resulted in preserved pigmentation.

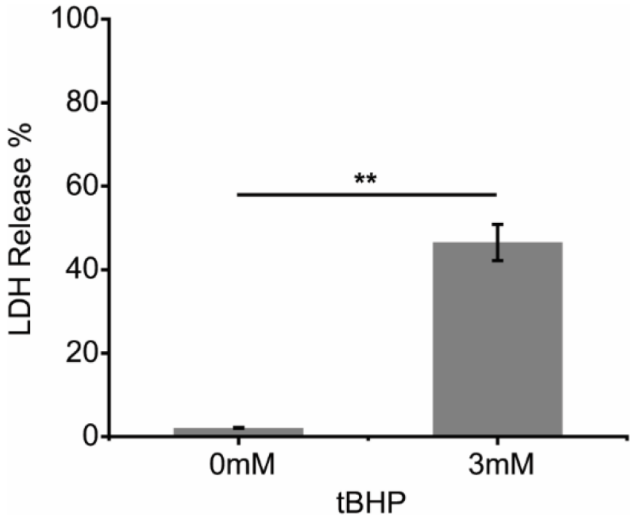

Supplementary Figure 5. Lactate dehydrogenase (LDH) assay accurately assesses cell death, as determined by exposure to the oxidant tert-butyl hydroperoxide (tBHP). Primary fetal RPE cultures were exposed to tBHP for 24 hours at a concentration known to cause partial but not complete cell death on the Transwell. There is a corresponding marked increase in LDH release. Data normalized to maximum possible LDH release as well as to the no tBHP condition.

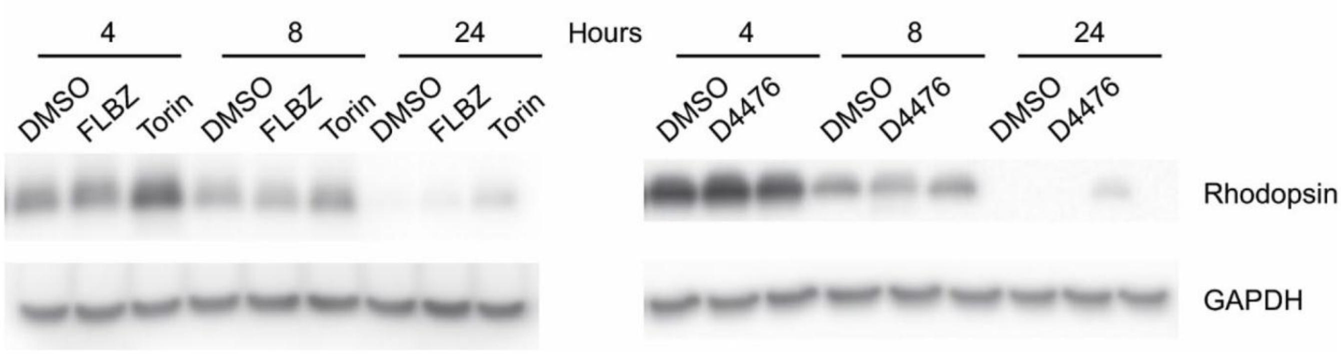

Supplementary Figure 6. Uncropped western blots for Figure 2. The phagocytosis blots for GSK in Figure 2C are uncropped and therefore do not appear above.
