# Supplementary material for: Pharmacologic activation of autophagy without direct mTOR inhibition as a therapeutic strategy for treating dry macular degeneration
Source: Aging (Albany NY). 2021 Apr 19;13(8):10866–90. doi: 10.18632/aging.202974 (PMC8109132; doi:10.18632/aging.202974)
Supplement: Supplementary Table 1 [file aging-13-202974-s002.pdf]

SUPPLEMENTARY TABLE

Supplementary Table 1. Commercial sources for autophagy inducers in this study.

Name	Abbreviation	Company	Product number
**GSK 1059615**	**GSK**	**Sigma-Aldrich**	**SML0083**
**Torin1**	**Torin**	**ApexBio**	**A8312**
**D4476**		**Cayman**	**13305**
**Flubendazole**	**FLBZ**	**Sigma-Aldrich**	**34091**
**Amiodarone**		**Cayman**	**15213**
**GW7647**		**Cayman**	**10008613**
**JNJ-47965567**	**JNJ**	**Cayman**	**21895**
2-Acetyl-5-tetrahydroxybutyl Imidazole	THI	Cayman	13222
Ac-Calpastatin		Cayman	16501
AZ-10606120		Tocris	3323
Bortezomib		Cayman	10008822
BRD5631		Broad Institute	MTA*
Carbamazepine		Sigma-Aldrich	C4024
Clonidine		Sigma-Aldrich	C7897
Entinostat	MS-275	Cayman	13284
Erlotinib		Cayman	10483
Fasudil	HA-1077	Cayman	10010559
Fenofibrate		Cayman	10005368
Fluphenazine		MP Biomedical	ICN15370691
K604		Otava LTD	7070707145
Loperamide		Cayman	14875
Metformin		Cayman	13118
ML246	Metarrestin	AOBious	AOB1384
Mocetinostat		Cayman	18287
Nilotinib		Cayman	10010422
Nilvadipine		Sigma-Aldrich	SML0945
Oxaprozin		Cayman	15476
Rilmenidine		Sigma-Aldrich	R134
Saroglitazar		Zydus-Cadila	MTA*
Sertraline		Cayman	14839
Spermidine		Sigma-Aldrich	S2626
Trifluoperazine		Sigma-Aldrich	T8516
(±)-Verapamil		Sigma-Aldrich	V4629

Direct mTOR inhibitors that induced autophagy in primary human fetal RPE cultures are highlighted in red. Compounds that do not directly inhibit mTOR but did induce autophagy in RPE cultures by both analysis of LC3 lipidation and LC3 puncta formation are highlighted in orange. Compounds that induced autophagy in RPE culture by only LC3 lipidation but not puncta formation are highlighted in green. All other compounds failed to induce LC3 lipidation in RPE cultures. *=Materials Transfer Agreement.
